# Systematic stereoscopic analyses for cloacal development: The origin of anorectal malformations

**DOI:** 10.1038/srep13943

**Published:** 2015-09-10

**Authors:** Daisuke Matsumaru, Aki Murashima, Junichi Fukushima, Syuhei Senda, Shoko Matsushita, Naomi Nakagata, Masayasu Miyajima, Gen Yamada

**Affiliations:** 1 Department of Developmental Genetics, Institute of Advanced Medicine, Wakayama Medical University, Wakayama, Japan; 2 Division of Reproductive Engineering, Center for Animal Resources and Development, Kumamoto University, Kumamoto, Japan; 3 Laboratory Animal Center, Institute of Advanced Medicine, Wakayama Medical University, Wakayama, Japan

## Abstract

The division of the embryonic cloaca is the most essential event for the formation of digestive and urinary tracts. The defective development of the cloaca results in anorectal malformations (ARMs; 2–5 per 10,000 live births). However, the developmental and pathogenic mechanisms of ARMs are unclear. In the current study, we visualized the epithelia in the developing cloaca and nephric ducts (NDs). Systemic stereoscopic analyses revealed that the ND-cloaca connection sites shifted from the lateral-middle to dorsal-anterior part of the cloaca during cloacal division from E10.5 to E11.5 in mouse embryos. Genetic cell labeling analyses revealed that the cells in the ventral cloacal epithelium in the early stages rarely contributed to the dorsal part. Moreover, we revealed the possible morphogenetic movement of endodermal cells within the anterior part of the urogenital sinus and hindgut. These results provide the basis for understanding both cloacal development and the ARM pathogenesis.

The cloaca is a transient embryonic cavity composed of endodermal epithelium and surrounding mesenchyme at the caudal end of the hindgut in most mammals. It has been described in medical textbooks that the urogenital sinus (UGS; ventral) and the rectum (dorsal) are formed as a result of cloacal separation^1^,^2^. Defective cloacal development and resultant anorectal and urogenital malformations are some of the most severe congenital anomalies in humans^3^. Anorectal malformations (ARMs) represent a common pathology that occurs in approximately 2–5 per 10,000 live births^4^. ARM phenotypes include a spectrum of symptoms from benign to severe (complex), such as perineal fistula, rectourethral fistula and cloaca. These symptoms are associated with several diseases such as Currarino syndrome, Townes-Brocks syndrome and VACTERL complex^5^,^6^,^7^,^8^,^9^. ARM phenotypes in the experimental models have been reported including the maternal administration of all-trans retinoic acid and the genetic mutants for Hedgehog, ephrin-Eph, Fibroblast growth factor and Wnt signaling-related genes^10^,^11^,^12^,^13^,^14^,^15^,^16^,^17^,^18^,^19^. These signaling molecules are suggested to regulate the development of several organs through epithelial-mesenchymal interactions^20^,^21^,^22^,^23^,^24^. In addition, analyses of human fetuses suggest that the spectrum of anomalies associated with cloaca malformation likely results from the timing of embryological developmental arrest^3^,^25^. Although these causative factors for ARMs have been pointed out, the pathogenic mechanisms remain unclear.

Several mechanisms have been proposed to explain the processes of cloacal division based on animal studies. One explanation is the fusion of the bilateral longitudinal folds (Rathke’s folds) for the formation of the urorectal septum (URS) and its “caudal movement” for the division of the cloaca into two compartments^26^,^27^,^28^. Another explanation is septation by a rostrally positioned fold (known as Tourneux’s fold: URS). It is believed that the URS divides the cloaca from the rostral to caudal direction^29^. Generally, many analyses have been basically performed in accordance with one or both of these models. In contrast to these two URS-based models, it has alternatively been proposed that cloacal division is achieved by descent of the dorsal cloaca to the tailgroove rather than the development of the URS^30^. Other researchers have suggested that the URS is formed passively as a result of cloacal development^31^,^32^. A recent analysis suggested that oriented cell division in the cloacal epithelium is essential for cloacal separation^33^. Moreover, the term “cloacal septation” itself has also been questioned^34^.

Although there have been several discussions on the development of the cloaca, stereoscopic analyses are rarely utilized. In the current study, we performed systematic three-dimensional reconstruction analyses of early staged embryos. The time-course analysis visualized the division processes of the cloaca. We observed the massive apoptosis and preserved basement membrane in the developing cloacal epithelium. In addition, a series of BrdU incorporation assays, including a pulse-chase analysis, suggested the presence of possible remodeling and different dorsoventral characteristics in the cloacal epithelium. These results may contribute to the understanding of the physiology of cloacal development, thereby providing the basis for the pathogenic mechanisms of ARMs.

## Results

### Cloacal division proceeds with a dynamic alteration of the epithelial morphology

**Figure 1:**
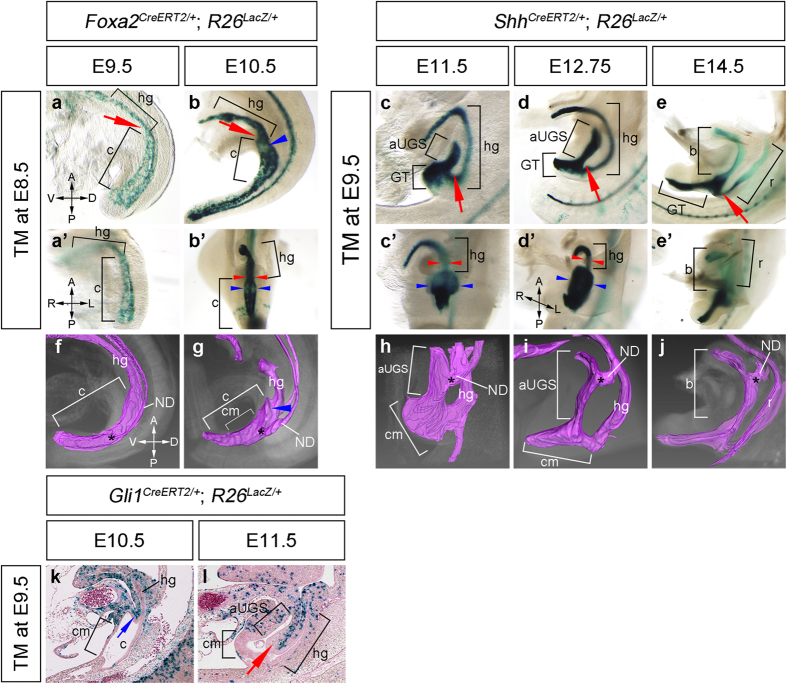
Three-dimensional analyses of cloacal division processes. X-gal stained *Foxa2*^*CreERT2*/+^; *R26*^*LacZ*/+^ embryos and *Shh*^*CreERT2*/+^; *R26*^*LacZ*/+^ embryos (**a**–**e**, **a’**–**e’**). Lateral view (**a**–**e**), frontal view (**a’**–**c’**) and frontal oblique view (**d’**, **e’**) of the embryos. *Foxa2*^*CreERT2*/+^; *R26*^*LacZ*/+^ embryos were treated with tamoxifen (TM) at E8.5 and harvested at E9.5 (**a**, **a’**) and E10.5 (**b**, **b’**). *Shh*^*CreERT2*/+^; *R26*^*LacZ*/+^ embryos were treated with TM at E9.5 and harvested at E11.5 (**c**, **c’**), E12.75 (**d**, **d’**), E14.5 (**e**, **e’**). A blue arrowhead in (**b**) indicates the prospective UGS. Red arrows in (**a**–**e**) indicate the boundary area between the cloaca and hindgut. The areas between blue and red arrowheads indicate the lateral width in the cloaca and hindgut, respectively (**b’**-**d’**). The three-dimensionally reconstructed endoderm from serial sections of embryos (**f**–**j**). A blue arrowhead in (**g**) indicates prospective UGS. Asterisks in (**f**-**j**) indicate the connection sites of NDs to the cloaca. The sagittal sections of *Gli1*^*CreERT2*/+^; *R26*^*LacZ*/+^ embryos, which were treated with TM at E9.5 and harvested at E10.5 (**k**) and E11.5 (**l**). A blue arrow in (**k**) indicates the LacZ-positive region in the URS. A red arrow in (**l**) indicates the LacZ-negative region in the URS. aUGS: anterior part of the urogenital sinus, b: bladder, c: cloaca, cm: cloacal membrane, GT: genital tubercle, hg: hindgut, ND: nephric duct, r: rectum, A: anterior, D: dorsal, L: left, P: posterior, R: right and V: ventral.

To analyze the morphological alteration of the endodermal structure during cloacal division, we utilized the *Foxa2*^*CreERT2*^ and *Shh*^*CreERT2*^ strains and the *Rosa26R* (*R26*^*LacZ*^) Cre reporter strain. These two Cre-driver strains possess the tamoxifen (TM)-inducible type of Cre recombinase in the *Forkhead box A2* (*Foxa2*) and *Sonic hedgehog* (*Shh*) loci, respectively^35^,^36^,^37^,^38^. *R26*^*LacZ/LacZ*^ females were crossed with *Foxa2*^*CreERT2*/+^ males and analyzed at E9.5 and E10.5 subsequent to TM treatment at E8.5 (Fig. 1a, a’, b, b’). ICR females were crossed with *Shh*^*CreERT2*/+^; *R26*^*LacZ/LacZ*^ males and analyzed at E11.5, E12.75 and E14.5 subsequent to TM treatment at E9.5 (Fig. 1c–e, c’-e’). Harvested embryos were stained with X-gal and cleared by the fructose-based pSeeDB method^39^,^40^. In the *Foxa2*^*CreERT2*/+^; *R26*^*LacZ*/+^ and *Shh*^*CreERT2*/+^; *R26*^*LacZ*/+^ embryos, the cloaca and hindgut were positive and the nephric ducts (NDs) were negative for LacZ expression. The lateral views of X-gal-stained embryos showed cloacal division processes including development of the “URS”, which is the unstained region between the anterior part of the UGS (aUGS; the prospective bladder part) and hindgut (Fig. 1b, c red arrows). The URS appeared to elongate from the proximal part of the umbilical cord toward the proximal and ventral parts of the genital tubercle (GT; Fig. 1c–e; red arrows) and contributed to the formation of the bladder and rectum structures (Fig. 1e)^15^,^41^. The cloaca was observed as a slightly enlarged structure compared to the hindgut tube at E9.5 (Fig. 1a). The cloaca-hindgut boundary was more apparent at E10.5 (Fig. 1b). The differences in the lateral width of the hindgut and cloaca were observed at this stage (Fig. 1b’; the width between the red arrowheads and blue arrowheads). The formation of the prospective UGS was initiated as the bilateral edges of the cloacal epithelium (Fig. 1b, b’, g; blue arrowheads). Hence, the initial features of cloacal division could be observed at approximately E10.5. Subsequently, cloacal division dramatically proceeded from E10.5 to E11.5 (Fig. 1c). The differences of the lateral width between the aUGS and hindgut were more apparent at E11.5 and later stages (Fig. 1c’–e’). Moreover, the aUGS was flattened dorsoventrally at E11.5, E12.75 and E14.5 (Fig. 1c–e).

To analyze the cloacal structure using computerized data, we next performed three-dimensional reconstruction from serial sections of embryos using the Amira. Similar to the results of the cleared X-gal stained embryos, we observed cloacal division processes (Fig. 1f–j; Supplementary Movies 1–3 for the cloacal structures at E9.5, E10.5 and E11.5, respectively). In addition to the cloaca, the epithelia of NDs were visualized. Of note, the ND-cloacal connection sites were dramatically altered from E9.5 to E14.5 (Fig. 1f–j; asterisks). At E9.5 and E10.5, the connection sites were observed at the lateral side of the middle part of the cloaca (Fig. 1f, g). These sites were located in the dorsal side of the aUGS at E12.75 and E14.5 (Fig. 1i, j). We also observed that the “URS mesenchyme” was indistinguishable from the adjacent mesenchyme, which is laterally positioned to the cloaca and hindgut, through the analysis of sequential serial sections during reconstruction (Supplementary Movie 4; serial sections of *Shh*^*CreERT2*/+^; *R26*^*LacZ*/+^ embryos at E11.5). Therefore, we examined *Gli1*^*CreERT2*/+^; *R26*^*LacZ*/+^ embryos to investigate the developmental origin of the URS mesenchyme (the sandwiched mesenchyme between the aUGS and hindgut epithelia). The *Gli1*^*CreERT2*/+^; *R26*^*LacZ/LacZ*^ males were crossed with ICR females. We collected *Gli1*^*CreERT2*/+^; *R26*^*LacZ*/+^ embryos at E10.5 and E11.5 subsequent to TM injection at E9.5, prior to the initiation of cloacal division. The sagittal sections of X-gal stained embryos were analyzed (Fig. 1k, l). Although many of the cells in the presumptive URS mesenchyme were positive for LacZ at E10.5 (Fig. 1k; blue arrow), the cells in the most ventral part of the URS were negative at E11.5 (Fig. 1l; red arrow). Instead, LacZ-positive cells were observed in the middle part of the URS mesenchyme (Fig. 1l). Judging from these sections, the “URS mesenchyme” appeared to be composed of the aUGS and hindgut mesenchyme including the cells derived from the LacZ-negative region. These results may suggest that cloacal division is achieved through remodeling of the cloacal epithelium and its adjacent mesenchyme rather than separation by the elongation of the URS.

### The connection sites of NDs to the cloaca shift from the lateral-middle to dorsal-anterior part of the cloaca/UGS epithelia during cloacal development

**Figure 2:**
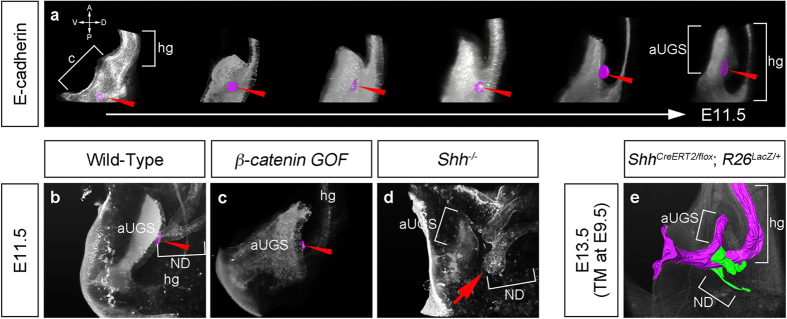
The cloacal morphology and connection sites of the nephric ducts. The endodermal structure was visualized using whole-mount immunofluorescence of E-cadherin (**a**–**d**). The embryonic cloacae in the wild-type embryos at approximately E10.5-11.5 were aligned by their morphology (**a**). To compare the general cloacal morphology, the magnifications in each embryo were not adjusted in (**a**). Lateral view of the wild-type (**b**), *β-catenin GOF* (**c**), and *Shh*^−/−^ (**d**) embryos stained for E-cadherin. The connection sites of NDs were marked with purple color and indicated by red arrowheads (**a**–**c**). A red arrow in (**d**) indicates an aberrant blind-ended ND. Three-dimensional reconstruction analyses revealed the abnormal cloacal morphology (marked with purple color) and abnormally positioned NDs (marked with green color) in *Shh*^*CreERT2/flox*^; *R26*^*LacZ*/+^ embryos treated with TM at E9.5. aUGS: anterior part of the urogenital sinus, c: cloaca, hg: hindgut, ND: nephric duct.

We further analyzed the embryos at E10.5 to E11.5 to investigate the alteration of the ND-cloaca connection sites during cloacal development. Embryos were stained by whole-mount immunofluorescence of E-cadherin antibodies and analyzed by confocal microscopy after tissue clearing. The urogenital parts of the embryos were aligned by their cloacal morphology (Fig. 2a). Serial confocal sections were processed using the Amira and the ND-cloaca connection sites were marked with purple color (Fig. 2a; red arrowheads). As cloacal development proceeded, the ND-cloaca connection sites altered from the lateral-middle side to the dorsal-anterior side of the cloaca/UGS.

We next analyzed the effects of cloacal malformation on the ND-cloaca connection sites. We utilized *β-catenin* gain-of-function mutants (*Shh*^*CreERT2*/+^; *β-catenin*^*flox(Ex3)*/+^, TM treatment at E9.5; hereafter described as *β-catenin GOF*) and *Shh*^−/−^ mutants, as these two strains have been shown to display ARM phenotypes^15^,^16^,^42^,^43^. β-catenin is an essential factor for the canonical Wnt pathway and it is also a component of the cadherin protein complex, which plays roles in stabilizing cell-cell contact^44^. Shh controls cell fate, cell proliferation, differentiation and tissue patterning during organognesis^11^,^45^. In addition to the previous observation of the defective elongation of URS^15^,^16^, the epithelial layer of the cloaca/UGS in *β-catenin GOF* mutants was abnormally enlarged and rounded (Fig. 2c). The ND-cloaca connection sites were observed in the dorsal-anterior part of the cloaca/UGS as observed in the wild-type embryos (Fig. 2c; red arrowhead). In contrast, *Shh*^−/−^ mutants displayed aberrant blind-ended ND epithelia in addition to cloacal hypoplasia at E11.5 (Fig. 2d; red arrow). Moreover, *Shh* conditional mutants (*Shh*^*CreERT2/flox*^; *R26*^*LacZ*/+^, TM treatment at E9.5) were analyzed at E13.5 to exclude the effects of cloacal malformation before the ND reaches the cloaca. X-gal stained embryos were sectioned and reconstructed using the Amira. LacZ-positive cloacal epithelium was marked with purple color and LacZ-negative NDs/ureters were marked with green color (Fig. 2e). At E13.5, the connection sites were positioned in the anterior and dorsal cloaca/UGS in wild-type embryos. In *Shh* conditional mutants, the NDs were connected to the posterior part of hypoplastic cloaca/UGS (Fig. 2e; Supplementary Movie 5). These results suggest the possibility that the cloacal structure may affect the ND connection sites.

### Cloacal division proceeds with massive cell death and preserved laminin expression

**Figure 3:**
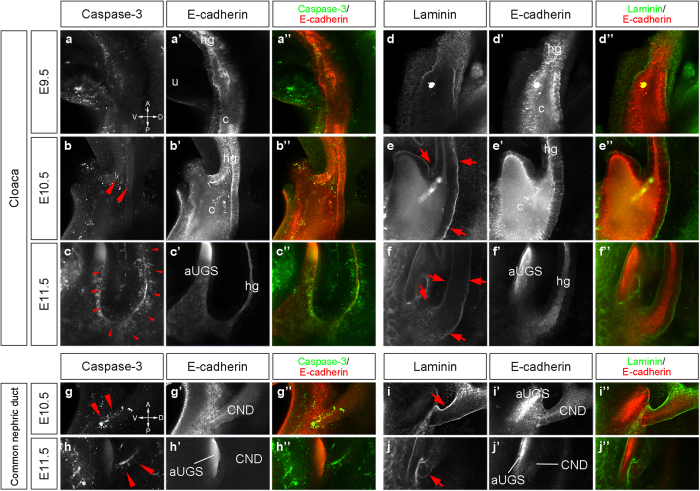
Whole-mount immunofluorescent analyses for the apoptotic cells and basement membrane structure in the cloaca and common nephric ducts. Whole-mount immunofluorescence of wild-type embryos at E9.5, E10.5 and E11.5. Sections were obtained by confocal microscopy. The embryos were stained with cleaved caspase-3 and E-cadherin antibodies (**a**–**c”**, **g–h”**) and laminin and E-cadherin antibodies (**d**–**f”**, **i**–**j”**). Cleaved caspase-3 expression was observed in both dorsal and ventral cloacal epithelia (red arrowheads). Laminin expression was observed on the basal side of the cloacal epithelium (red arrows). The CNDs were positive for cleaved caspase-3 and laminin expression (**g**–**j”**). Red arrowheads indicate cleaved caspase-3 expression and red arrows indicate laminin expression. aUGS: anterior part of the urogenital sinus, c: cloaca, CND: common nephric duct, hg: hindgut, u: umbilical region.

During cloacal division, drastic alteration of the cloacal morphology was observed from E10.5 onward (Figs 1a–e and 2a). It has been known that cell death is observed in the epithelium and middle part of the mesenchyme in the URS^41^,^46^. However, less information is available for the spatiotemporal cell death status at early stages. Hence, we examined the expression of cleaved caspase-3, an apoptotic cell marker, in the wild-type embryos at E9.5, E10.5 and E11.5. At E9.5, cleaved caspase-3 positive cells were not observed around the cloaca, but were observed in the umbilical region (Fig. 3a–a”). The cloacal epithelial cells were positive for cleaved caspase-3 at approximately E10.5 (Fig. 3b–b”; red arrowheads). Its expression was broadly distributed in the dorsal part of the aUGS and hindgut epithelia at E11.5 (Fig. 3c–c”; red arrowheads). The expression in the hindgut epithelium at E11.5 was mainly observed in the posterior to the upper ridge of the aUGS. Three-dimensional confocal analyses revealed that cleaved caspase-3 positive cells were distributed around the dorsal part of the aUGS and hindgut epithelia at E11.5 and the lateral width in the hindgut part was narrower than that in the aUGS (Supplementary Movie 7).

We next analyzed the cellular mechanisms underlying cloacal epithelial development. The morphology of the cloacal epithelium was dramatically altered with a substantial number of cleaved caspase-3 positive cells at approximately E11.5. Generally, for the maintenance of the epithelial structure displaying abundant cell death, cells can be supplied from the adjacent epithelial layer or adjacent mesenchyme. We then analyzed the expression of laminin, a marker of the basement membrane. Using whole-mount immunofluorescence, it was revealed that the expression of laminin was retained in the cloacal/UGS epithelia at E9.5, E10.5 and E11.5 (Fig. 3d–f; red arrows). These results excluded the possibility of cell supply from the mesenchyme to the apoptotic epithelium via processes including the mesenchyme-to-epithelial transition.

The common part of the ducts between the Wolffian duct and ureter, referred to as the common nephric duct (CND), also displays extensive cell death (Fig. 3g, h)^47^,^48^. We then analyzed the expression of these markers in the CND region. The expression of laminin was also observed in the CND at E10.5 and E11.5 (Fig. 3i, j; red arrows). Based on a previous report which demonstrated that the CND does not differentiate into the trigone^47^, these findings suggest that the CND epithelium is shortened by apoptosis while the basement membrane is maintained.

### The ventral epithelial cells in the cloaca/UGS may regulate cloacal division

**Figure 4:**
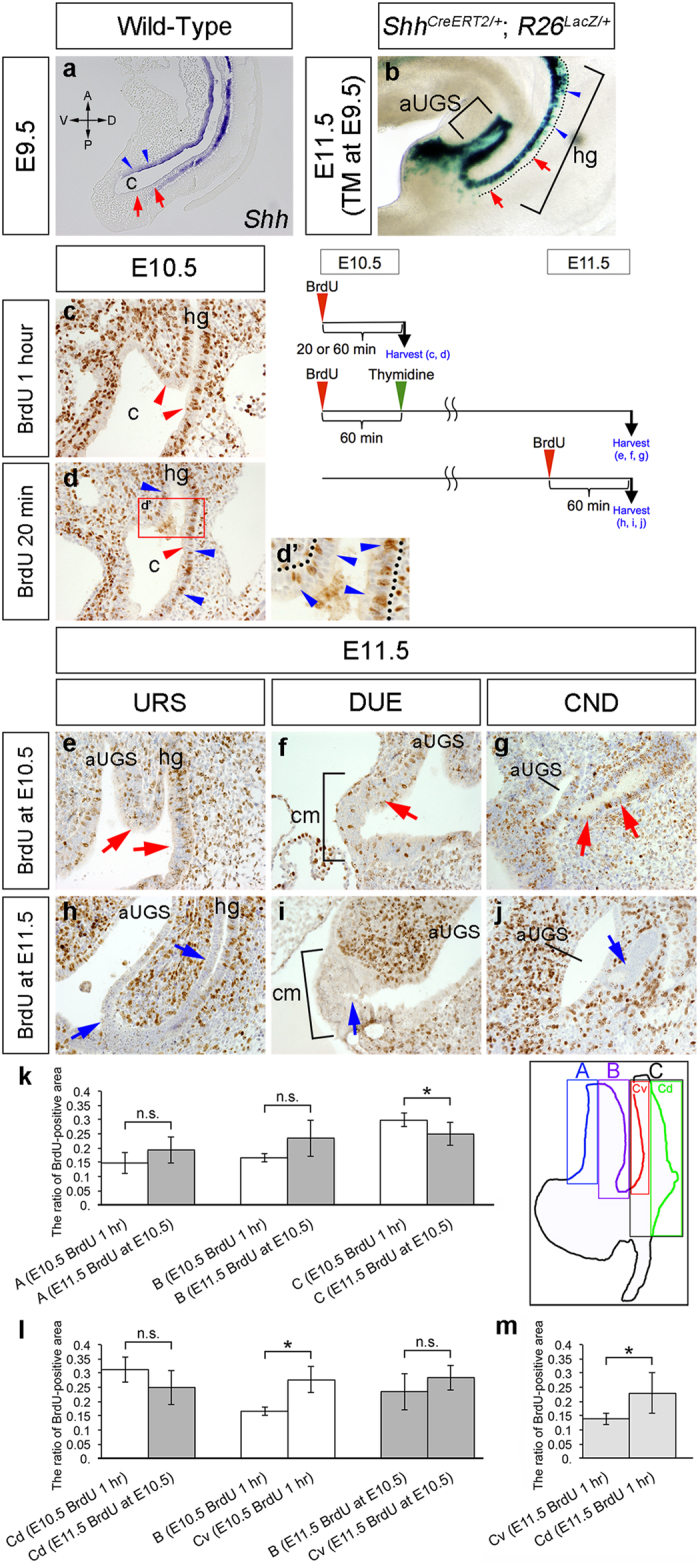
Dynamic distribution of the BrdU-positive cells and dorsoventral polarity of the cloacal epithelium. The expression of *Shh* mRNA in the sagittal section of E9.5 wild-type embryos (**a**). The lateral view of X-gal stained *Shh*^*CreERT2*/+^; *R26*^*LacZ*/+^ embryos at E11.5 following TM administration at E9.5 (**b**). Blue arrowheads indicate the *Shh*- or LacZ-positive cells and red arrows indicate the *Shh*- or LacZ-negative cells in the cloacal epithelium. The wild-type embryos at E10.5 were treated with BrdU for 1 hour (**c**) or 20 minutes (**d**). The demarcated area in (**d**) is magnified in (**d’**). The wild-type embryos at E11.5 were treated with thymidine 1 hour after BrdU treatment at E10.5 (**e**–**g**) or treated with BrdU for 1 hour (**h**–**j**). Embryos were stained with anti-BrdU antibodies (**c**–**j**). Red arrowheads in (**c**) and (**d**) indicate the BrdU-positive cells in the cloacal epithelium. Blue arrowheads in (**d**) and (**d’**) indicate BrdU-positive cells on the basal side of the cloacal epithelium. Section immunohistochemistry of BrdU in the URS, distal urethral epithelium (DUE) and CND regions at E11.5 subsequent to BrdU treatment at E10.5 (**e**–**g**; red arrows) or 1 hour treatment of BrdU at E11.5 (**h**–**j**; blue arrows). The cloacal epithelium was divided into 5 areas: A: ventral cloacal epithelium excluding the cloacal membrane, B: dorsal cloacal epithelium, C: hindgut and dorsal cloacal epithelia, Cv: ventral part of C and Cd: dorsal part of C. The ratios of BrdU-positive areas in the defined epithelial area were compared between E10.5 embryos (BrdU for 1 hour) and E11.5 embryos (BrdU at E10.5) (**k**). The comparison of the ratios between E10.5 embryos and E11.5 embryos in Cd (**l**; left). The comparisons between areas B and Cv in E10.5 embryos (BrdU for 1 hour) (**l**; middle) or in E11.5 embryos (BrdU at E10.5) (**l**; right). The comparison between areas Cv and Cd in E11.5 embryos (BrdU for 1 hour) (**m**). The results of the statistical analyses in (**k**–**m**) are showed in Supplementary Table. The results are presented as the means ± SD. *P < 0.05 (**k**–**m**). aUGS: anterior part of the urogenital sinus, c: cloaca, cm: cloacal membrane, hg: hindgut.

The dorsoventral patterning of the cloacal epithelium has been discussed recently. It was reported that Sox2-positive cells were detected at the dorsal side of the cloacal epithelium at E10.5^49^. Despite these regional differences in marker expression, the temporal fate of the cloacal epithelial cells is not well understood. Hence, we analyzed gene expression in the cloacal epithelium. The expression of *Shh* was observed only in the ventral hindgut and cloacal epithelia in the early stages, such as at E9.5 (Fig. 4a). Its expression was uniformly observed in the cloacal epithelium at E10.5 and later stages^15^,^50^. Thus, the contribution of ventral cloacal epithelial cells can be analyzed through genetic labeling of Shh-expressing cells at approximately E9.5. We analyzed *Shh*^*CreERT2*/+^; *R26*^*LacZ*/+^ embryos. A small dosage of TM was administered at E9.5 to achieve a temporally restricted genetic labeling of Shh-expressing cells. The LacZ-expressing cells were mainly observed in the ventral UGS and hindgut epithelia at E11.5 (Fig. 4b). This result suggests that the cells located on the ventral cloacal/hindgut epithelia rarely contribute to the dorsal part. The present stereoscopic analysis revealed that the initial stage of cloacal division involves the folding of the ventral cloacal epithelium (Fig. 1). Hence, it is suggested that the ventral epithelial cells contribute to cloacal division.

A biased pattern of cell proliferation is occasionally observed during organogenesis, such as in the case of intestinal crypts and ureteric buds^51^,^52^. We therefore analyzed the cell proliferation status during cloacal development. First, wild-type embryos were treated with BrdU at E10.5 and harvested after 20 minutes or 1 hour of incubation. Following BrdU incubation for 20 minutes or 1 hour, BrdU-positive cells were observed in the cloacal epithelium and mesenchyme (Fig. 4c, d). In general, short-term BrdU labeling results in a restricted incorporation pattern compared to long-term exposure in the highly proliferative tissues. We expected the presence of proliferating cells in the “URS tip region” for effective septation. Despite the expectation, BrdU-positive cells in the prospective tip of the URS region were not abundantly observed following short-term labeling for 20 minutes (Fig. 4d). Of note, the BrdU-positive cells in the epithelial layer were significantly concentrated on the basal side in short-term treated embryos (Fig. 4d, d’; blue arrowheads).

Cloacal division proceeds with massive cell death and preserved basement membrane in the cloacal epithelium (Fig. 3c–c”, f–f”). To investigate the contribution of proliferating epithelial cells from E10.5 to E11.5, a BrdU pulse-chase analysis was performed. The distal urethral epithelium (DUE) is an endoderm-derived epithelial area located at the distal tip of the GT^43^,^53^. Apoptotic cells are present in the DUE and the epithelium of the CND^43^,^47^,^48^,^53^. In addition to the URS epithelium, we therefore analyzed these epithelial regions to examine the possible similarities and differences of both of these characteristics. We injected thymidine 1 hour after BrdU injection at E10.5 to terminate the incorporation of BrdU. Subsequently, embryos were collected at E11.5 (24 hours after BrdU injection) (Fig. 4e–g). BrdU-positive epithelial cells were observed in the URS, DUE and CND (Fig. 4e–g; red arrows). In contrast, fewer epithelial BrdU-positive cells were observed in the embryos treated with BrdU for 1 hour at E11.5 compared to the number of cells in the adjacent mesenchyme (Fig. 4h–j). To interpret the results of the pulse-chase analysis, quantitative analyses were performed. According to possible regional differences in the cloacal epithelial character, we divided the cloacal epithelium into five parts: A: ventral cloacal epithelium excluding the cloacal membrane, B: dorsal cloacal epithelium, C: hindgut and dorsal cloacal epithelia, Cv: the ventral part of C and Cd: the dorsal part of C. The ratios of BrdU-positive areas in the defined epithelial areas were then analyzed using the ImageJ. We first compared the E10.5 embryos (BrdU for 1 hour) and E11.5 embryos (BrdU at E10.5) (Fig. 4k). There were no significant differences in areas A and B. In area C, the ratio in E10.5 embryos (BrdU for 1 hour) was significantly higher than that in E11.5 embryos (BrdU at E10.5). Since the BrdU-positive cells (Fig. 4c-g) and cleaved caspase-3 positive cells (Fig. 3a-c”) tended to be distributed randomly in each part at these stages, we hypothesized the presence of morphogenetic movement, such as cellular migration, inside the epithelial layers. The BrdU-positive ratios in the area Cd between these two groups were not significantly different (Fig. 4l; left). This result prompted us to analyze the area Cv. We then analyzed the comparisons between areas B and Cv in each group of embryos. The embryos at E10.5 (BrdU treatment for 1 hour) displayed a significant difference of BrdU-positive ratios between areas B and Cv (Fig. 4l; middle). This tendency was not observed in E11.5 embryos (BrdU at E10.5) (Fig. 4l; right). These results suggest the presence of morphogenetic movement from area Cv to B from E10.5 to E11.5. Moreover, we observed a significant difference of the ratios of BrdU-positive areas between areas Cv and Cd in E11.5 embryos (BrdU for 1 hour) (Fig. 4m). This result further suggests the possibly different characteristics between ventral and dorsal epithelia in the hindgut part of the cloaca.

### The stereoscopic visualization of the cloacal epithelia reveals morphological abnormality and altered distribution of apoptotic cells in Shh^−/−^ and β-catenin GOF mutants

**Figure 5:**
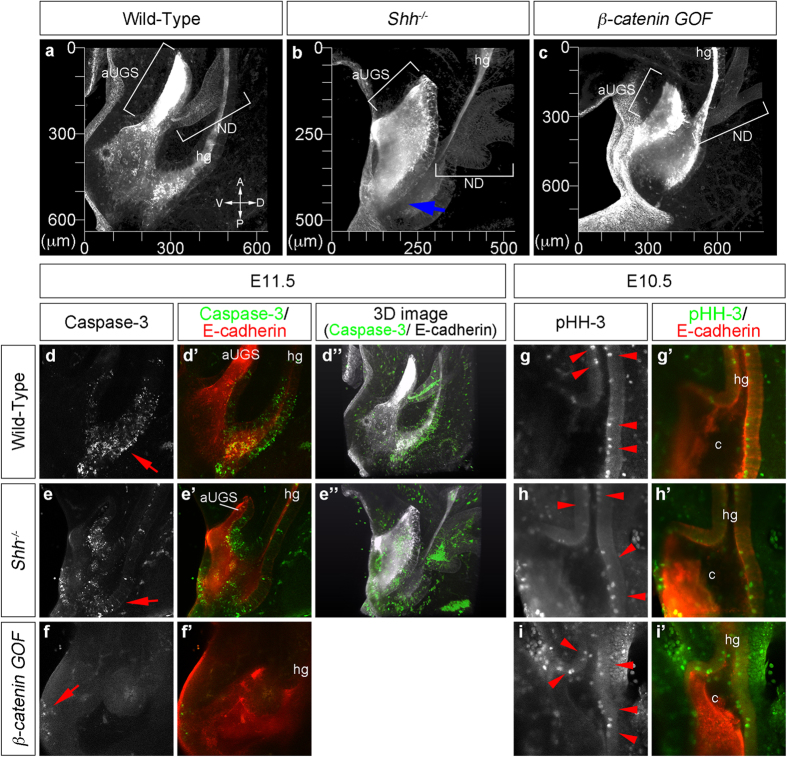
Stereoscopic analyses for the morphology, apoptosis and cell proliferation in the cloacal epithelium. The wild-type, *Shh*^−/−^ and *β-catenin GOF* embryos were immunohistochemically stained with E-cadherin antibodies (**a**–**c**). A blue arrow in (**b**) indicates the abnormally elongated lateral edge of the UGS. The wild-type, *Shh*^−/−^ and *β-catenin GOF* embryos stained with cleaved caspase-3 and E-cadherin antibodies at E11.5 (**d**–**f’**). Red arrows indicate cleaved caspase-3 positive cells in the cloacal epithelium (**d**, **e**) and DUE (**f**). A three-dimensional view of E-cadherin (white color) and cleaved caspase-3 (green color) in wild-type (**d”**) and *Shh*^−/−^ (**e”**) embryos. The wild-type, *Shh*^−/−^ and *β-catenin GOF* embryos stained with pHH-3 and E-cadherin antibodies at E10.5 (**g**–**i’**). Red arrowheads indicate pHH-3 positive cells in the apical part of the cloacal epithelium. aUGS: anterior part of the urogenital sinus, c: cloaca, hg: hindgut, ND: nephric duct.

Cloacal organogenesis, including its division, proceeds dynamically and three-dimensionally. The previous analyses on the developmental mechanisms have been performed mostly by normal histological analyses. We therefore analyzed the morphology of cloacal epithelium and the distribution of apoptotic cells in the mutants exhibiting ARM phenotypes: *Shh*^−/−^ and *β-catenin GOF* (*Shh*^*CreERT2*/+^; *β-catenin*^*flox(Ex3)*/+^)^15^,^42^. The embryos were stained with E-cadherin antibodies and analyzed three-dimensionally (Fig. 5a–c; Supplementary Movies 6–8). Stereoscopic analyses revealed the abnormal shape of the cloaca/UGS in these mutants (Fig. 5a–c). Namely, the shape of the aUGS in the *Shh*^−/−^ mutants was hypoplastic and the lateral edges of the UGS epithelium were abnormally elongated to the posterior part in the *Shh*^−/−^ cloaca (Fig. 5a,b; blue arrow). In addition, GT protrusion was defective (Fig. 5b). The shape of ND epithelium was also abnormally enlarged in the *Shh*^−/−^ mutants (Fig. 5b). In *β-catenin GOF* mutants, the hypoplastic and unflattened aUGS were observed (Fig. 5c; Supplementary Movie 6). Moreover, the distribution of apoptotic cells during cloacal division was also assessed in these mutants. Cleaved caspase-3 expression was observed in the URS region of wild-type and *Shh*^−/−^ mutants at E11.5 (Fig. 5d, e; red arrows; Supplementary Movies 7, 8). Its expression in the dorsal epithelium of the aUGS was broader in the *Shh*^−/−^ compared to the wild-type embryos (Fig. 5d, e; Supplementary Movies 7, 8). In *β-catenin GOF* mutants, cleaved caspase-3 expression in the URS region was reduced^15^, but retained in the DUE (Fig. 5f; red arrow). The URS epithelium became thicker in the *β-catenin GOF* mutants in comparison to the wild-type embryos (Fig. 5f, f’). The three-dimensional analysis further revealed the spatial distribution of cleaved caspase-3 positive apoptotic cells in the NDs, aUGS and hindgut epithelia of wild-type and *Shh*^−/−^ embryos (Fig. 5d”, e”; Supplementary Movies 7, 8).

We next performed whole-mount immunofluorescence of phospho-histone H3 (pHH-3) for detecting proliferating cells in wild-type, *Shh*^−/−^ and *β-catenin GOF* embryos at E10.5 (Fig. 5g–i, g’–i’). The distribution of pHH-3 positive cells in the epithelial layer was comparable among all embryos. It is noted that the pHH-3 positive cells were observed on the apical side of the epithelial layer in wild-type embryos (Fig. 5g, g’; red arrowheads). The apical distribution of pHH-3 positive cells (M-phase) and the basal distribution of BrdU-positive cells (S-phase) following short-term treatment (Fig. 4d, d’) indicated alterations of apico-basal nuclear location depending on the cell cycle status in the cloacal epithelium. This differential location of the cells in the epithelial layer is in agreement with the mode of interkinetic nuclear migration (INM). In *Shh*^−/−^ and *β-catenin GOF* mutants, pHH-3 positive cells were also observed on the apical side of the epithelial layer (Fig. 5h, i, h’, i’; red arrowheads). These results suggest that ARM phenotypes observed in these mutants are not due to defective INM at early stages.

## Discussion

The division of the embryonic cloaca is an essential event for the formation of digestive and urinary outlets. Defects in this process are one of the major causes for anorectal malformations (ARMs) in several syndromic diseases^5^,^6^,^7^,^8^,^9^. However, our understanding of the mechanisms for the division of the cloaca is not sufficient due to the lack of detailed three-dimensional analyses. In the present study, we analyzed cloacal division using systematic stereoscopic methods. From the combination of these analyses and histological analyses, it is suggested that the URS mesenchyme is composed of the mesenchyme of the aUGS and hindgut. The epithelial cells in the developing cloaca possess dorsoventral characteristics in the early stages and the cells in the ventral part of the cloaca may play roles in cloacal division. These results shed light on the understanding of the mechanisms of cloacal development and the pathogenesis of ARMs.

Several mechanisms have been proposed to explain cloacal division. One explanation is the fusion of bilateral folds (Rathke’s folds) from a rostral to caudal direction, like a zipper^26^,^27^. This model has been questioned by a few observations regarding the fusion of the bilateral folds^31^. Another mechanism is the elongation of the URS (Tourneux’s fold)^29^. Cloacal septation by the URS is thought to be the simplest explanation for the division. Many experiments have been performed in accordance with this model. This model has also been questioned and other potential explanations include the cloaca-remodeling model (e.g., the shift of the dorsal cloaca to the tailgroove region)^30^,^33^. The shift of the dorsal cloaca model is based on the presence of the URS structure, regardless of its antero-posterior length. The present analysis showed that the URS structure emerges at approximately E10.5 as a fold of the ventral endodermal epithelium in mouse embryos. This may be due to the loss of the diverticulum connecting the allantois at this stage, which was not detected in the lateral view of endodermally LacZ-expressing embryos. Therefore, careful explanations for murine cloacal division would be necessary. In the present analysis, we observed a wider lateral width of the cloaca than that of the hindgut. Generally, the width of two divided regions derived from one common region would be expected to be similar. During current three-dimensional analyses, the URS mesenchyme was indistinguishable from the mesenchyme of the aUGS and hindgut. It is therefore more appropriate to suggest that the URS in the sagittal plane is composed of the mesenchyme of the aUGS and hindgut. This observation is not contradictory to the descriptions in medical textbooks which indicate that the URS is formed by the fusion and wedging of the mesoderm surrounding the allantois and the hindgut^1^,^2^. Moreover, through the analysis of *Gli1*^*CreERT2/+*^; *R26*^*LacZ*/+^ embryos, we also showed that parts of the cells in the “elongating URS” were not positive for LacZ, regardless of its expression in the early stage. This result suggests that some parts of the URS mesenchyme are derived from a LacZ-negative mesenchyme. Hence, our findings support alternative explanations for cloacal division, rather than the elongation of fold(s)-mediated models such as Rathke’s folds and Tourneux’s fold.

According to the present analyses, we classify cloacal division into three steps: (1) the initiation of cloacal division with the folding of the ventral part of the endodermal epithelium at the boundary between the prospective aUGS and hindgut, (2) separation of the UGS and hindgut with a substantial number of apoptotic epithelial cells and (3) opening of the rectum on the basal part of the elongating GT.

Cloacal division initially starts with the specification of the aUGS by the folding of the ventral epithelia between the prospective aUGS and hindgut together with the formation of the bilateral epithelial edges of the aUGS. It has been reported that the transcription factor Sox2 was specifically expressed in the dorsal cloacal epithelium at E10.5^49^. Such regionalization of the cloacal epithelium has not yet been well studied. The present study examined the contribution of the ventral cloacal epithelial cells in the early stages to the later cloaca/UGS. The analysis of *Shh*^*CreERT2*/+^; *R26*^*LacZ*/+^ embryos revealed that the ventrally located cells in the early stage rarely contributed to the dorsal part during cloacal division. Moreover, the current statistic BrdU pulse-chase analysis suggests the possible morphogenetic movement of epithelial cells, such as migration, from the hindgut to the aUGS. These observations suggest the possibility that ventral epithelial cells play central roles for the initial cloacal division. Although *Shh*^−/−^ and *β-catenin GOF* (*Shh*^*CreERT2*/+^; *β-catenin*^*flox(Ex3)*/+^) embryos displayed ARM phenotypes in the later stages, the initiation of the folding of the endodermal epithelium in the prospective boundary between the UGS and hindgut was comparable to those of the wild-type embryos. This suggests that the initial step of cloacal division is independent of *Shh* and *β-catenin* signaling. In the present study, we also observed apico-basal localization of the epithelial cell nuclei depending of the cell cycle status, which is referred to as interkinetic nuclear migration (INM). The suggested functions of INM include effective packaging of cells to the epithelium and regulation of the epithelial shape in the hinge points by the differential location of nuclei^54^,^55^. It has also been suggested that cell division at the apical surface may be important for generating forces to shape the epithelium^56^. The current mutants displaying ARM phenotypes showed apical distribution of pHH-3 positive epithelial cells, which was comparable to that of the wild-type embryos. Hence, it is suggested that INM is also independent of *Shh* and *β-catenin* signaling. Further analysis such as the effects of factors regulating INM for the cloacal epithelium will reveal the contribution of INM for initial cloacal development.

Subsequent to the above folding of the ventral epithelium between the aUGS and hindgut, cloacal division proceeds to separate the UGS and hindgut with a substantial number of apoptotic cells. The present study showed that the epithelia in the dorsal cloacal/UGS and common nephric ducts (CNDs) displayed massive apoptosis^41^,^46^,^47^,^48^ while preserving laminin expression during their development. It has been previously shown that there are no contributions of the CND epithelium to the bladder trigone through a genetic lineage analysis utilizing *Hoxb7*^*Cre*^; *R26*^*LacZ*^ embryos^47^,^57^. Hence, it can be expected that the reduced level of apoptosis may lead to an abnormal persistence of these structures. In the dorsal cloacal epithelium, defective apoptosis was accompanied by the defective division of cloaca in *β-catenin GOF* mutants. The *β-catenin GOF* mutants displayed an unflattened aUGS and a larger lateral width of the dorsal cloaca than that of the control embryos. These observations suggest that massive apoptosis in the cloacal epithelium contributes to cloacal morphogenesis. These results suggest the differential effects of epithelial apoptosis between the dorsal cloaca and CND. Moreover, a higher incorporation of BrdU in E11.5 embryos was observed in the dorsal part of cloacal epithelium compared to the ventral part. It has been suggested that asymmetric characteristics of cell proliferation and gene expression are essential in several embryogenesis processes^58^,^59^. It is therefore important to investigate how these characteristics of cloacal epithelium are established and determine whether these characteristics promote the separation of the UGS and hindgut during cloacal division as a second process of its development.

In the latter stages, cloacal division may be affected by the protrusion and elongation of the GT. The protrusion of the GT leads to the appropriate positioning of the cloacal membrane. Abnormal shortening and/or rotation of the cloacal membrane are suggested as causative factors for ARM pathogenesis^19^,^31^. Moreover, previous reports suggested that a ventral shift of the dorsal cloaca from E11.5 to E12.5 accompanies the development and growth of the GT^41^. The present analysis utilized two types of ARM mutants. One of the differences between *Shh*^−/−^ and *β-catenin GOF* mutants is the defective protrusion of the GT in the *Shh*^−/−^ mutants. In the *Shh*^−/−^ mutants at later stages, the prospective URS reaches around the cloacal membrane^42^,^43^,^50^. Therefore, the abnormal cloacal division in the *Shh*^−/−^ mutants is suggested to be derived from abnormal GT formation. In fact, several mutants with GT defects including *p63*^−/−^, *Wnt5a*^−/−^ and *β-catenin* cKO embryos also display urogenital abnormalities^13^,^15^,^19^,^60^,^61^. On the other hand, the *β-catenin GOF* mutants of the present study displayed the perineal cleft and hypospadias-like phenotypes in addition to the cloaca/UGS phenotypes^15^. Since the mesoderm in the URS can contribute to the GT^62^, the phenotype in the GT might occur as a result of abnormal cloacal division. These observations suggest that GT formation and cloacal division may influence each other.

In the present study, we suggested the presence of possible remodeling and dorso-ventral differences of the cloacal epithelium. According to the present findings, cloacal division can be classified into three steps: (1) cloacal division begins with the folding of the ventral part of the endodermal epithelium at the prospective boundary between the UGS and hindgut together with the formation of the bilateral epithelial edges of the aUGS; (2) the division proceeds to separate the UGS and hindgut with a substantial number of apoptotic cells. During this process, the hindgut reduces in lateral width; (3) the rectum opens on the outer surface of the proximal GT possibly through the effects of the protrusion of the GT. Along with the above model, it is suggested that the lack of apoptosis and defective GT protrusion together with a hypoplastic endodermal structure, may lead to the ARM phenotypes observed in *β-catenin GOF* and *Shh*^−/−^ mutants, respectively.

The extrapolation from animal model studies to human disease is essential to advance our understanding of the human pathogenesis. The Krickenbeck Classification divides ARMs according to the type of the fistula in addition to rare and regional variants^63^. Both male and female types of fistula are considered to be as derived from abnormalities in formation of the cloaca and/or the URS^1^,^2^. These causative factors might correspond to “Steps (1) and (2)” in the current model. As for the other types of fistula, the perineal fistula involves a misplaced anal opening which is located outside of the sphincter muscle complex. A previous report suggests that the cloacal/perineal muscles, including the anal sphincter, form by the extension from the ventral muscle mass of the hindlimb region^64^. This observation suggests that abnormalities in the destination of the muscle precursors and/or the position of the anal opening may result in a perineal fistula. Hence, this may be partially related to “Step (3)” in the current model. We believe that the current observations will contribute to a better understanding of cloacal development and ARM pathogenesis.

## Methods

### Mice

The mutant mice used in this study were *Foxa2*^*CreERT2*^*, Shh*^*CreERT2*^, *Gli1*^*CreERT2*^, *R26*^*LacZ*^, *β-catenin*^*flox(Ex3)*^, *Shh*^*flox*^ and *Shh*^−/−^ 35,36,45,65,66,67,68. For *Foxa2*^*CreERT2*/+^; *R26*^*LacZ*/+^ embryos, *R26*^*LacZ/LacZ*^ females were crossed with *Foxa2*^*CreERT2*/+^ males and administered 1.5 mg per 30 g maternal body weight of TM. For *Shh*^*CreERT2*/+^; *R26*^*LacZ*/+^ embryos, ICR (Jcl:ICR, CLEA Japan, Osaka, Japan) females were crossed with *Shh*^*CreERT2*/+^; *R26*^*LacZ/LacZ*^ males and administered 3 mg per 30 g maternal body weight of TM. A small dosage (0.5 mg per 30 g maternal body weight) of TM was utilized to achieve ventrally restricted labeling of *Shh*^*CreERT2*^. For *Gli1*^*CreERT2*/+^; *R26*^*LacZ*/+^ embryos, ICR females were crossed with *Gli1*^*CreERT2*/+^; *R26*^*LacZ/LacZ*^ males. The mouse embryos were processed for X-gal or immunohistochemical staining. Noon of the day on which the vaginal plug appeared was designated as embryonic day 0.5 (E0.5). All experimental procedures and protocols were approved by the Committees on Animal Research at Wakayama Medical University and Kumamoto University and the experiments were carried out in accordance with the approved guidelines.

### Histological Analyses

The mouse embryos were fixed overnight in 4% paraformaldehyde with PBS, dehydrated with methanol and embedded in paraffin. Subsequently, 6-μm serial sections were prepared for RNA *in situ* hybridization analyses. The *Shh* riboprobe utilized in this study was kindly provided by C. Shukunami (Kyoto University, Japan). X-gal staining was carried out as previously described^50^. The X-gal stained samples were cleared using the pSeeDB method^39^,^40^ or embedded in paraffin and sectioned. For systematic three-dimensional reconstruction analyses, serial sections of X-gal stained embryos were processed using the Amira 5.6 software program. Whole-mount immunofluorescent analyses were performed according to the procedures on the Abcam website (http://www.abcam.co.jp/). The dilution ratios of primary antibodies were as follows: anti-E-cadherin (1:500, BD), anti-cleaved caspase-3 (Asp175) (1.25:500, Cell Signaling), anti-laminin (1.25:500, Sigma-Aldrich) and anti-phospho-histone H3 (Ser10) (pHH-3) (1:500, Millipore). For secondary antibodies, appropriate Alexa antibodies were utilized (2:500, Molecular Probes). The stained samples were cleared using the pSeeDB method and processed by confocal microscopy^39^,^40^. For the cell proliferation assays, pregnant females were injected with 100 mg of 5-bromo-2’-deoxyuridine (BrdU, Sigma-Aldrich) per kg of body weight. One hour after injection, the embryos were collected and processed for immunohistochemistry with anti-BrdU antibodies (1:300, Roche). For the BrdU pulse-chase analysis, 10 mg of thymidine was administered 1 hour after BrdU injection.

### Statistical analysis

For the statistical analyses of the ratio of BrdU-positive areas, the defined epithelial area and BrdU-positive area were measured using the ImageJ 1.44 software program. The ratio of these areas was calculated and statistical significance was analyzed using Student’s *t*-test or Welch’s alternate *t*-test for equal or unequal variances. A probability of less than 0.05 was considered to indicate statistical significance.

## Additional Information

**How to cite this article**: Matsumaru, D. *et al.* Systematic stereoscopic analyses for cloacal development: The origin of anorectal malformations. *Sci. Rep.* **5**, 13943; doi: 10.1038/srep13943 (2015).

## Supplementary Material

Supplementary Information

Supplementary Movie 1

Supplementary Movie 2

Supplementary Movie 3

Supplementary Movie 4

Supplementary Movie 5

Supplementary Movie 6

Supplementary Movie 7

Supplementary Movie 8
