# Supplementary material for: Systematic stereoscopic analyses for cloacal development: The origin of anorectal malformations
Source: Sci Rep. 2015 Sep 10;5:13943. doi: 10.1038/srep13943 (PMC4564729; doi:10.1038/srep13943)
Supplement: Supplementary Information [file srep13943-s1.pdf]

Systematic stereoscopic analyses for cloacal development: The origin of anorectal malformations

Daisuke Matsumaru[1,2], Aki Murashima[1,2], Junichi Fukushima[1], Syuhei Senda[1], Shoko Matsushita[1], Naomi Nakagata[3], Masayasu Miyajima[4], Gen Yamada[1,2]*

[1]Department of Developmental Genetics, Institute of Advanced Medicine, Wakayama Medical University, Wakayama, Japan; [2]Institute of Molecular Embryology and Genetics, Kumamoto University, Kumamoto, Japan; [3]Division of Reproductive Engineering, Center for Animal Resources and Development, Kumamoto University, Kumamoto, Japan; [4]Laboratory Animal Center, Institute of Advanced Medicine, Wakayama Medical University, Wakayama, Japan

Supplementary Information

Supplementary Table. The statistical analyses of BrdU-positive cell ratios.

The figures indicate the results of the statistical analyses from Figure 4k-m.

Supplementary Movie 1. The three-dimensionally reconstructed cloaca at E9.5.

Supplementary Movie 2. The three-dimensionally reconstructed cloaca at E10.5.

Supplementary Movie 3. The three-dimensionally reconstructed cloaca at E11.5.

Supplementary Movie 4. Sequential serial sections of *Shh*$^{CreERT2/+}$; *R26*$^{LacZ/+}$ embryos at E11.5.

Supplementary Movie 5. The three-dimensionally reconstructed cloaca of *Shh*$^{CreERT2/flox}$; *R26*$^{LacZ/+}$ mutants at E13.5.

Supplementary Movie 6. The three-dimensional confocal image of the cloaca in *Shh*$^{CreERT2/+}$; *β-catenin*$^{flox(Ex3)/+}$ mutants stained with E-cadherin antibodies at E11.5.

Supplementary Movie 7. The three-dimensional confocal image of the cloaca in wild-type embryos stained with E-cadherin and cleaved caspase-3 antibodies at E11.5.

Supplementary Movie 8. The three-dimensional confocal image of the cloaca in *Shh*$^{-/-}$ mutants stained with E-cadherin and cleaved caspase-3 antibodies at E11.5.

Supplementary Table.

Figure 4k

	A (E10.5 BrdU 1 hr)	A (E11.5 BrdU at E10.5)	B (E10.5 BrdU 1 hr)	B (E11.5 BrdU at E10.5)	C (E10.5 BrdU 1 hr)	C (E11.5 BrdU at E10.5)
Sample size	5	5	5	5	5	5
Mean	0.14847	0.19362	0.16633	0.23524	0.29953	0.24939
SD	0.03739	0.0459	0.01448	0.06402	0.0236	0.04098
SEM	0.01672	0.02053	0.00648	0.02863	0.01056	0.01833
Two-tailed distribution p-level	0.12649		0.07875		0.04516 (P < 0.05)	

Figure 4l

	Cd (E10.5 BrdU 1 hr)	Cd (E11.5 BrdU at E10.5)	B (E10.5 BrdU 1 hr)	Cv (E10.5 BrdU 1 hr)	B (E11.5 BrdU at E10.5)	Cv (E11.5 BrdU at E10.5)
Sample size	5	5	5	4	5	5
Mean	0.31268	0.24975	0.16633	0.27643	0.23524	0.28454
SD	0.04451	0.06024	0.01448	0.04618	0.06402	0.04377
SEM	0.01991	0.02694	0.00648	0.02309	0.02863	0.01957
Two-tailed distribution p-level	0.0971		0.00139 (P < 0.05)		0.19293	

Figure 4m

	Cv (E11.5 BrdU 1 hr)	Cd (E11.5 BrdU 1 hr)
Sample size	5	5
Mean	0.13917	0.22866
SD	0.02047	0.07147
SEM	0.00915	0.03196
Two-tailed distribution p-level	0.04322 (P < 0.05)	
